# Remote sensing and disease control in China: past, present and future

**DOI:** 10.1186/1756-3305-6-11

**Published:** 2013-01-11

**Authors:** Zhijie Zhang, Michecal Ward, Jie Gao, Zengliang Wang, Baodong Yao, Tiejun Zhang, Qingwu Jiang

**Affiliations:** 1 Department of Epidemiology, School of Public Health, Fudan University, Shanghai 200032, People’s Republic of China; 2 Key Laboratory of Public Health Safety, Ministry of Education, Shanghai 200032, People’s Republic of China; 3 Laboratory for Spatial Analysis and Modeling, School of Public Health, Fudan University, Shanghai 200032, People’s Republic of China; 4 Faculty of Veterinary Science, The University of Sydney, Camden, NSW, Australia

**Keywords:** Remote sensing, Satellites, Disease control, Human health, China

## Abstract

Satellite measurements have distinct advantages over conventional ground measurements because they can collect the information repeatedly and automatically. Since 1970 globally and 1985 in China, the availability of remote sensing (RS) techniques has steadily grown and they are becoming increasingly important to improve our understanding of human health. This paper gives the first detailed overview on the developments of RS applications for disease control in China. The problems, challenges and future directions are also discussed with an aim of guiding prospective studies.

## Introduction

Remote sensing (RS) is the acquisition of information about an object or phenomenon on Earth, without directly making physical contact. In modern usage, RS generally refers to the use of aerial sensor technologies to detect and classify objects (both on the earth surface and in the atmosphere and oceans) by means of propagated signals such as electromagnetic radiation emitted from aircraft or satellites [1-3]. Based on RS images, a lot of information on detected objects can be obtained. This includes information on environmental factors (e.g., vegetation, land use/land cover, and water bodies), which are closely related with the occurrence of many environment-related diseases and can affect their distributions accordingly. So various extracted factors from RS can serve as a bridge for linking RS techniques with disease studies. However, there is a prerequisite for these kinds of studies-there must be a close association between life-cycle variables for the diseases of interest (or their intermediate host species) and environmental features that can be extracted from RS images [4].

As early as 1970, Cline highlighted the prospects for using aerial pictures and other RS techniques in epidemiological research [5]. In 1971, the National Aeronautics and Space Administration (NASA) established the Health Applications Office (HAO) to organize and coordinate collaborations and programs on applying RS techniques in disease studies and considerable pioneering work was undertaken in the late 1970s. In July 23, 1972, the first Earth Resources Technology Satellite (ERTS-1) was successfully launched (it was soon to be renamed Landsat 1), signaling a historical breakthrough in technology to apply RS techniques in studies of human health. Twelve years later, the first RS applications to predict the occurrence of human schistosomiasis in the Caribbean and the Philippines were published [6,7]. Nowadays, RS techniques has been widely adopted and applied in various fields of health such as viral diseases (e.g., Dengue fever [8] and Rift valley fever [9]), bacterial diseases (e.g., Plague [10], Lyme disease [11]), and parasitic diseases, including schistosomosis [12], malaria [13], trypanosomosis [14], leishmaniasis [15], filariasis [16], dracunculosis [17] and echinococcosis [18]. Several reviews of RS applications in health have previously been undertaken, but they have either focused on a specific disease or concentrated on the joint applications of RS, geographic information systems (GIS) and spatial data analysis technologies [19-25]. Very few have specifically reviewed the applications of RS techniques on disease control and none in China.

The aim of this review is to discuss the progress made to date in the field of disease control in China in which RS is a major research tool, to identify problems in current and previous studies, and to highlight the challenges, the prospects and directions for future studies applying RS techniques on disease control.

### Search strategy for literature review

The NCBI PubMed ( http://www.ncbi.nlm.nih.gov/pubmed/) and CNKI ( http://www.cnki.net/, one of the biggest biomedical literature databases in China) were used to search the related English and Chinese literature for application of RS techniques in disease studies of China. The search terms were “((remote sensing [Title] OR satellite [Title]) AND China) AND English [Language]” and “(remote sensing [Title] OR satellite [Title])”, respectively, and the search period was limited to January 30, 2012.

We first skimmed through the abstracts of the searched papers and only those papers closely related to applying RS in disease control in China were included. Then, manual searches of the cited references in the selected literature were conducted and relevant articles were retained.

## Review

### Stage 1 (1985–1995): Introducing the ideas of RS techniques for disease control

During this period, Chinese researchers began to realize and think about the possibility of applying RS for disease control.

In 1985, the first paper on using RS images (Landsat MSS) to identify the habitats of snails, the sole intermediate host of schistosomiasis, was published [26], indicating the commencement of a discussion of applying RS techniques for disease control in China. Unfortunately, in that paper the authors only mentioned that they used RS images in their work, but did not describe details of the methods of processing and analyzing the images. This may explain why it did not attract more attention from other researchers. Five years later, a second study identifying different ecological zones of snails using RS images (Landsat MSS and Airborne RS) through the approach of manual visual interpretation was reported with many details on how to analyze the RS images [27]. Since then, Chinese researchers began to realize the potential of applying RS for disease control, and started to introduce and study the successful experiences on utilizing RS in disease control from abroad, with most of the work reviewing the international developments in that direction.

Gu reviewed the developments in using RS images in the surveillance of mosquito breeding habitat (e.g., *Aedes* and *Anopheles*) [28]; Chen and Hu discussed the possibilities of applying RS images (Landsat and National Oceanic and Atmospheric Administration (NOAA) satellite sensor) to study endemic diseases (e.g., schistosomiasis, fluorosis, keshan and kaschin beck diseases) from the viewpoint of geo-ecological zones [29]; while Wang, Wang and Liao, and Lin summarized the international developments of utilizing RS images in the field of epidemiology [30-32].

### Stage 2 (1996–2003): Exploration of various RS analysis techniques

Researchers in China began to investigate the feasibility of ideas and methods to analyze and utilize RS images for disease control activities (Table 1).

**Table 1:** Characteristics of studies using RS techniques in disease control studies during 1996-2003

**Disease**	**Study area**	**Study aim**	**RS**	**Spatial analysis**	**Reference**
schistosomiasis	South of the Yellow River	To explore the possibility of using prediction model for schistosomiasis surveillance.	NOAA-AVHRR, 1 km	Overlay analysis	[33]
schistosomiasis	Dantu county, Zhenjiang city, Jiangsu province	To quantitatively measure the changes of marshland area related to schistosomiasis.	Aerial photography maps	Manual measurement	[34]
schistosomiasis	Anning River, Xichang city, Sichuan province	To determine whether environmental conditions observable via Landsat TM imagery correlate with the presence of snails.	Landsat TM, 30 m	Unsupervised and supervised classification	[35]
schistosomiasis	Yangtze River within Nanjing city, Jiangsu province	To understand the distribution of snail habitats in the lake and marshland regions.	Landsat MSS, 30 m	Tasseled Cap Transformation	[36-38]
schistosomiasis	Liupo village, Guichi region, Anhui province	To identify the suitable vegetation types for snails.	Landsat TM, 30 m	Unsupervised classification	[39]
schistosomiasis	Poyang Lake	Identify the water regions in schistosomiasis epidemic regions.	Landsat TM, 30 m	Visual interpretation	[40]
schistosomiasis	Poyang lake	To identify snail habitats in Poyang Lake regions.	Landsat TM, 30 m	Unsupervised classification	[41]
Malaria	Jiangsu Province	To explore the possibilities of predict the trend of malaria epidemic with RS images.	NOAA-AVHRR, 1 km	Correlation analysis	[42]
schistosomiasis	Jiangning county	To explore the relationship of NDVI and snail habitats.	NOAA-AVHRR and MODIS Terra, 1 km	Linear regression	[43]
Dengue fever	Guangdong province	To explore the relationship of NDVI and *Aedes* density.	NOAA-AVHRR, 1 km	Linear regression	[44]

Zhou et al. assessed the possibility of applying a climate-based parasite forecast model to predict the risk region for schistosomiasis transmission, where the composite map of normalized difference vegetation index (NDVI) from the Advanced Very High Resolution Radiometer (AVHRR) satellite sensor was used as a background map to overlay with the map of schistosomiasis transmission index [33]; Sun et al. calculated the fluctuations of the marshland area in the section of the Yangtze River within Dantu county in Jiangsu Province during 1973–1987 by manually measuring the area on the aerial photography maps [34]. Spear et al. applied a two-tiered classification approach (ISODATA clustering and maximum likelihood algorithm) on the Landsat TM images to identify the snail habitats in Anning River in Xichang city of Sichuan province for determining the relationships between RS-derived environmental conditions and the presence of snails [35]. In 1999, Zhou et al. used the tasseled-cap transformation indices to extract the wetlands in Landsat MSS images as the “potential” snail habitats and compared the dynamics of the habitats among three different seasons of the catastrophic flood season (August 1983), the annual flood season (August 1984), and the dry season (March 1984) [36]. This was later adapted by using Landsat TM images to study the dynamics of snail habitats along the section of the Yangtze River in Nanjing and Yangzhou cities, respectively [37,38]. Lin et al. used principal component analysis to select the appropriate Landsat TM bands (TM3, TM4 and TM5 were chosen) for unsupervised classification and then the classes suitable for snails were determined through a field survey [39,45,46]. In contrast, Jiang et al. focused on identifying water bodies in the regions of schistosomiasis epidemics through pseudo-color composition and visual interpretation [40].

In 2002, Yang et al. explored the possibilities of applying NOAA-AVHRR images to predict the epidemic trends of malaria in Jiangsu Province by establishing the relationship of malaria incidence and NDVI using the method of correlation analysis [42], which was then adopted to study the association between malaria prevalence in Hainan province and the NDVI from the NOAA-AVHRR [47]. Meanwhile, the relationship between snail habitats and NDVI, and the relationship between *Aedes* density and NDVI were studied by Zhang et al. [43,48,49] and Yi et al. [44], respectively, using similar approaches of correlation analysis. These studies began to apply the statistical analysis techniques in the process of utilizing RS images, signaling the appearance of another direction for using RS images in disease control-integrating RS-extracted environmental indices into the framework of spatial statistical modeling.

### Stage 3 (2003-present): Two directions of RS applications for disease control

During this period, two major research directions based on different notions of utilizing RS images were continuously developed. One direction was identifying the appropriate environments for the intermediate hosts or vectors of certain diseases through in-depth study on the classification techniques of RS images (Table 2). Another direction was predicting the appropriate environments for the occurrence of a disease of interest and the related intermediate hosts and vectors through spatial modeling techniques with RS-extracted environmental factors as explanatory variables (Table 3).

**Table 2:** Characteristics of studying classification techniques of RS images for disease control during 2003-present

**Disease**	**Study area**	**Study aim**	**RS**	**Spatial analysis**	**Reference**
schistosomiasis	Dongzhi county, Anhui province	To explore appropriate index for monitoring snail habitats.	Landsat TM, 30 m	Unsupervised classification	[50]
schistosomiasis	Jiangning county	To analyze the vegetation characteristics of snail habitats.	Landsat ETM+, 30 m	Unsupervised classification	[51]
schistosomiasis	Poyang Lake	To identify snail habitats.	Landsat TM, 30 m	Unsupervised classification and tasseled-cap transformation	[52]
schistosomiasis	Zhongxiang city,Hubei province	To identify snail habitats.	Landsat TM, 30 m	Neural network analysis	[53]
schistosomiasis	Poyang lake	To identify snail habitats.	Landsat TM, 30 m	Knowledge-based Decision trees	[54]
schistosomiasis	Guichi region, Anhui province	To identify snail habitats.	CBERS, 20 m	Index-based quantitative classification	[55]
schistosomiasis	Poyang lake	To predict the distribution of snail habitats.	Landsat TM, 30 m	Fuzzy classification	[56]
schistosomiasis	Dali city, Yunnan province	To predict the suitability of snail habitats.	Landsat TM, 30 m	Suitability modeling technique	[57]
plague	Tongyu county, Jilin province	To identify appropriate regions for the living of *Spermophilus dauricus*.	Landsat TM, 30 m	Unsupervised classification	[58]

**Table 3:** Using RS-extracted environmental indices as covariates in the process of spatial data modeling

**Disease**	**Study area**	**Study aim**	**RS**	**Spatial analysis**	**Reference**
schistosomiasis	Jiangning county	To predict snail density.	Landsat ETM+, 30 m	Linear regression analysis and Kriging interpolation	[59]
schistosomiasis	Xichang city, Sichuan province	To predict snail density.	Ikonos, 4 m; ASTER, 30 m	Linear regression and semi-variogram analysis	[60]
schistosomiasis	Jiangsu province	To study the spatio-temporal variation of schistosomiasis infection risk.	NOAA-AVHRR, 1 km	Bayesian spatial modeling	[61]
malaria	Southeastern Yunnan Province	To study the relationship of RS-extracted NDVI to Anopheles density and malaria incidence rate.	NOAA-AVHRR, 1 km	principal component analysis, factor analysis and grey correlation analysis	[62]
schistosomiasis	Jiahu village of Yugan county (Poyang Lake)	To study quantitative relationships between snail density and various environmental indices from RS images.	Landsat TM, 30 m	Linear regression analysis	[63]
schistosomiasis	Eryuan county, Yunnan Province	To understand ecological variability of snail distribution.	SPOT5, 5 m	Bayesian spatial modeling	[64]
schistosomiasis	Guichi region, Anhui province	To identify the risk regions of schistosomiasis.	NOAA-AVHRR, 1 km; CBERS, 20 M	Generalized additive models	[65]

#### Continuing studies on the classification techniques of RS images

Zhang et al. studied the possibility of using different environmental indices (NDVI, green vegetation index (GVI), brightness index (BI)) from Landsat TM images to monitor snail habitats and found that NDVI/GVI can be used to identify snail habitats and predict the possible ranges of snail dispersal [50]. Zhang et al. used the unsupervised classification technique to analyze the vegetation characteristics of snail habitats by first filtering the regions without vegetation coverage using NDVI from Landsat ETM + images [51,66]. Guo et al. used a two-step method to identify snail habitats indicated by the two important ecological characteristics of snail habitats, “land in winter and water in summer” and “no snails if no grass”. The former was obtained by subtracting the water regions (unsupervised classification) in the dry season image from the wet season image, while the latter was obtained using NDVI and tasseled-cap transformation indices (brightness, greenness and wetness index) to extract the regions with vegetation coverage in the dry season. Then overlaying the two layers-water difference regions and vegetation coverage-the intersecting regions satisfying the above two features were defined as potential snail habitats [52]. The same approach was adopted by Yang et al. to predict the potential habitats of *Oncomelania hupensis* in the Hongze, Baima and Gaoyou lakes in Jiangsu province [67]. This method was believed to be a good approach for identifying the potential habitats suitable for snails, but we noted that they also used the environmental indicators from RS images to extract the regions with vegetation coverage and applied the method of unsupervised classification to identify the water regions related to snail habitats. To improve this approach, Zhang et al. first introduced the normalized difference water index (NDWI) to the field of schistosomiasis research and modified the above method by using the NDWI index from RS images to quantitatively extract the water regions for snail habitats [55,65], which was later shown to be a better method [68-70].

Besides, some new methods for classifying RS images were also investigated during this period. For example, Niu et al. explored artificial neural networks (ANNs) to classify the Landsat TM images for detecting snail habitats [53]. Zhang et al. used the knowledge-based decision tree to classify Landsat TM images for detecting snail habitats [54]. Zhao and Bao predicted the spatial distribution of snail habitats based on the combined datasets of Landsat TM images and GIS thematic data (e.g., digital elevation model (DEM), soil and land use) through the approach of knowledge-driven fuzzy classification [56]. Dong et al. applied the suitability modeling technique on the indices of NDVI, LST, wetness and land use to predict the suitability of snail habitats and classified the regions into three types of unsuitable, suitable and optimum snail environments [57].

While Ju et al. first applied the approach of unsupervised classification on Landsat TM images to identify the possible regions able to support *Spermophilus dauricus*, the major host of plague in China [58].

#### Spatial modeling techniques with RS-extracted indices as covariates

Zhang et al. extracted various environmental indices from Landsat ETM + images, including modified soil-adjusted vegetation index (MASVI), land surface temperature (LST), tasseled-cap transformation indices (brightness, greenness, and wetness indices) and then modeled their relationships with snail density by joint application of linear regression model and kriging interpolation techniques [59]. Xu et al. applied a similar analysis strategy to model snail density with land-cover and land-use fractions in the mountainous regions of schistosomiasis [60]. Liu and Chen evaluated the relationship between NDVI (from NOAA-AVHRR), rainfall and air temperature and *Anopheles* density and malaria incidence rate via principal component analysis, factor analysis and grey correlation analysis. They found that NDVI is a sensitive index for assessing disease associations [62]. Gao et al. established quantitative relationships between snail density and RS-extracted indices, including NDVI, GVI, global environment monitoring index (GEMI), perpendicular vegetation index (PVI) and second modified soil adjusted vegetation index (MASVI2), using traditional linear regression models [63].

Yang et al. studied the spatio-temporal variation of schistosomiasis japonicum infection risk in Jiangsu province, China using the climatic factors (NDVI and LST) extracted from RS images as covariates in Bayesian models [61]. This is the first spatial study in the real sense by simultaneously taking into account spatial autocorrelation and predictors in the models. Yang et al. modeled the relationship between snail density of schistosomiasis in mountainous regions and environmental surrogates (NDVI) and landscape metrics (e.g., mean shape index (MSI), Shannon’s evenness index (SEI), slope, proportion of paddy fields, and proportion of agrarian roads) from high-resolution SPOT5 images (5 m in panchromatic mode and 10 m in colored mode) to understand the ecological variability of the distribution of *Oncomelania hupensis* at the local scale using the Bayesian techniques [64,71]. Zhang et al. presented a systematic approach for locating the active transmission site (ATS) of schistosomiasis- “the high-risk snail habitats where infected snails are frequently present and with which people are often in contact” [65,72]. The snail habitats were first extracted based on two indices of NDVI and NDWI suggested by [55,65,68-70]; then the relationships between the schistosomasis data and the potential risk factors (e.g., RS-derived environmental indices) were modeled and the high-risk regions of schisotosmiasis were further identified. Finally, 6 ATS were located by overlaying the above detected high-risk regions of schistosomiasis and the snail habitats extracted from RS images [65]. This is a promising approach for sustainable control of schistosomiasis.

#### Problems and challenges of applying RS in disease control

It has been nearly 30 years since the beginning of studies applying RS techniques for disease control in China, and great developments have been made. From the above review, we can identify four characteristics in the application of RS techniques for disease control in China:

(1) Started late, but developed rapidly. Chinese researchers recognized the possibility of using RS in disease control in 1985, approximately fifteen years later than other international researchers (1970). But closer international collaborations have resulted in a fast pace to catch up with the recent progress.

(2) RS applications were mainly at the low (e.g., 1KM NOAA-AVHRR) and medium resolutions (e.g., 20 M China-Brazil Earth Resources Satellite (CBERS), 30 M Landsat TM). The high spatial resolutions (such as QuickBird and SPOT images) are rarely used and microwave imagery is absent, which may be caused by the high cost of obtaining those images.

(3) From the early simple analysis methods to the latest research approaches. In the very beginning, researchers only were able to use the basic unsupervised or supervised classification algorithm on RS images to detect different objects on the earth, but now many modern methods (such as the fuzzy classification technique and artificial network analysis) have been combined into the process of RS classification and spatial data modeling.

(4) RS applications have been extended from single diseases to multiple diseases (e.g., malaria, Dengue fever, Plague), but most of the RS studies (>90%) in disease control are still on schistosomiasis-the most serious parasitic disease in China.

The achievements in using RS in disease control are obvious, but exciting developments are sparse. Here some key problems and challenges are highlighted, which will be discussed with schistosomiasis and malaria as examples.

##### Factors influencing diseases are complicated

Snails are the sole intermediate host of schistosomiasis, hence most RS studies are on snail habitats because its distribution is consistent with that of schistosomiasis to a great degree. It needs to be recognized that the presence of snail habitats is just a necessary condition for schistosomiasis, not a sufficient condition [73]. But the actual studies are always simplified such as only vegetation and water factors are considered. Such studies could be greatly improved by overlaying distributions of other conditions such as land-cover/land-use data, elevation, land forms, hydrological network, soil properties, and even human activity patterns and cattle grazing patterns [74].

##### Using the presence of vector or host to predict the disease distribution

Snails are the sole intermediate host of schistosomiasis, so the distribution of schistosomiasis is always indicated by snail habitats, which can be detected through the RS-extracted environmental conditions. But there may be two limitations in such studies [75]: a) there are only fundamental correlations between the snail habitats and schistosomiasis, so the direct causal relationship linking environmental conditions to vector distribution or abundance remains to be established; b) schistosomiasis risk is more closely related to the abundance of infected snails, rather than the simple presence of snails, or total abundance of snails. To differentiate the positive snail habitats from negative ones is more important, and the integrated two-step modeling framework proposed by Zhang et al. [65] might be promising, but more effective approaches to discriminate snail habitats with infected snails from non-infected snails deserve further research.

##### Using disease incidence to estimate disease risk

In recent years, there has been increasing interest in integrating RS-extracted variables within the process of spatial data modeling to identify high-risk regions of diseases. The disease incidence or prevalence is used to estimate disease risk but there are distinct discrepancies between risk and disease occurrence (incidence or prevalence). For instance, the widespread use of preventative measures for malaria (e.g., mosquito bednets or filtration of drinking water) can strongly reduce the disease incidence, but the risk is still high [76]. For those diseases, four types of region with different implications for disease control could be created: high incidence and high risk, high incidence and low risk, low incidence and high risk, and low incidence and low risk. This distinction has been completely ignored.

##### RS applications are employed only for limited diseases in China

RS techniques have been widely used for many diseases around the world, such as Lyme disease, paracoccidioidomycosis, ebola fever, hantavirosis, Saint-Louis encephalitis, Rift Valley fever, West Nile virus, dracunculiasis, echinococcosis, fascioliasis, filariasis, leishmaniasis, malaria, trypanosomiasis, schistosomiasis and *Vibrio cholera*[77-80]. But in China over 90% of the RS studies are still on schistosomiasis, more potential diseases have yet to be explored [81,82].

##### Time-series dynamic disease studies using RS have not been explored

Current RS studies on human health in China are only targeted at detecting the spatial distribution of different objects, such as snail habitats, which are closely related with the disease of interest (e.g., schistosomiasis). The temporal dynamics of the objects determining the disease distribution has been rarely discussed.

##### Lack of the necessary skills and good coordination among different departments

Public health practitioners always have difficulties in accessing the latest RS images and the related environmental materials, and there is a lack of effective intersectoral collaboration. With respect to high resolution RS images, the costs are too high for routine applications. More importantly, extracting the information from RS images and applying it in studies of disease control needs multidisciplinary techniques such as geography, RS, biology, ecology, computer science, and so on, which are beyond the abilities of most ordinary users or even groups.

#### Future directions of using RS in disease control

##### New RS image processing methods should be extensively investigated for disease control

In the past, only the traditional RS classification approaches (e.g., supervised and unsupervised classification) were widely studied, while the application of many promising and novel methods, such as support vector machine [83], spatial data mining [84], neural network analysis [85], and object-based RS analysis techniques [86,87], have not been explored.

##### Adding the time-dimension to RS technique applications for human health

All previous RS researches for human health in China have been space-based static studies and have not considered the attribute of time, which may be important for monitoring and forecasting studies. For example, Linthicum et al. built an autoregressive integrated moving average (ARIMA) model combining both sea surface temperature and NDVI derived from AVHRR data to forecast the outbreak of rift valley fever five months in advance [88]. This can be sufficient lead time for decision-makers to take preventive measures and shows encouraging prospect for disease control.

##### Assessing RS data quality and evaluating their impact on disease control studies

As applied users, we only care about how to obtain RS data and how to use it for disease control. As such, we make one key assumption that the obtained RS data is reliable. Very few have ever thought about the issues of RS data quality, although data quality in spatial studies is critical. A comparison of two different sources of NDVI data sets for Africa (FAO-ARTEMIS and NASA Pathfinder AVHRR) revealed significant discrepancies, which may be caused by different methods of processing, such as the correction method of atmospheric effects. The authors concluded that these dataset are not directly inter-changeable [89]. Nowadays, many different types of RS data can be used either freely or at low costs, but whether the study results will change (or even reverse) if different RS data was used, how big is the difference, how to adjust for the differences and so on, have not been studied.

##### Extending the kinds of diseases that can be studied by RS techniques

In China, only vector-borne diseases have been explored by RS techniques, and schistosomiasis is the most widely studied disease. Extending the experiences and methods of applying RS techniques from vector-borne disease studies to other studies such as water- and soil- borne diseases is very meaningful and should be conducted as early as possible.

##### RS images with high spatial resolutions should be explored

Presently, RS images with high spatial resolutions are very expensive. This has prohibited their wide usage and conventional applications. But this situation is changing. In July 1988, China and Brazil signed the protocol establishing the joint research and production of the China-Brazil Earth Resources Satellites (CBERS). The first satellite of the series, CBERS-1, was successfully launched on October 14, 1999, and after that another four satellites were successfully launched in sequence, including CBERS-2, CBERS-2B, CBERS-2C, and CBERS-3. CBERS-1 and CBERS-2 are identical satellites with three remote sensing multispectral cameras: Wide Field Imager Camera (WFI) with 260 m spatial resolution, Medium Resolution Camera (CCD) with 20 m spatial resolution, and Infrared Multispectral Scanner Camera (IRMSS) with 80 m spatial resolution; CBERS-2B and CBERS-2C are similar to the two previous members of the series, but a new camera was added: High Resolution Panchromatic Camera (HRC) with around 2.5 m spatial resolution; while the newest satellite, CBERS-3, contains four cameras: Advanced Wide Field Imager Camera (AWFI) with 60 m spatial resolution, IRMSS with 40 m spatial resolution, Panchromatic and Multispectral Camera (PANMUX) with 5 m spatial resolution for the panchromatic band and 10 m spatial resolution in the other bands. For any researchers, the CBERS images with spatial resolution ≥20 m can be available freely through the CBERS website, but the high spatial resolution images such as 2.5 m must be applied by signing some official documents with CBERS institution and then can be obtained freely. For example, in 2012 our research group has signed a contract with the CBERS ( http://www.cresda.com/n16/index.html) for using RS images with 2.5 m resolution freely and unlimitedly. This will possibly become the trend for distributing high resolution RS images in the future (at least for academic researchers). So exploring their potential benefits for disease control is necessary.

##### Air pollution and human health

Since the low earth orbit Television Infrared Observation Satellite (TIROS-1) was launched on April 1, 1960, many satellites have been operated to provide earth observing information, which includes the measurement of land, ocean, clouds, radiation, and trace gases (e.g., SO_2_, NO_2_, CO, O_3_ and Aerosol optical depth (AOD) derived PM2.5/PM10) [90]. Besides, the natural hazards such as volcanoes can also be observed and monitored through RS images. Without the need for personnel to conduct on-site observations, the endangerment to human life has been minimized. The wide availability of environment- and climate-related data derived from RS images has stimulated a new and promising research direction, studying the relationships between RS-based estimates of air pollution and human health. For example, Evans et al. evaluated the global mortality attributable to particulate air pollution measured from RS images [91] and Anderson et al. studied the satellite-based ambient air pollution and global variations of childhood asthma prevalence [92].

## Conclusions

Pathogens use many different modes, such as direct contact (e.g. pathogens transmitted during aggressive or sexual encounters), near-direct contact (e.g. pathogens excreted by one host and inhaled or consumed by another), or relying on an arthropod vector, to disperse from infected to uninfected hosts. In most cases, the probability of transmission will decline dramatically with distance from an infected host. Hence, the factors affecting the spatial positions of pathogens, hosts and vectors, and their probability of close encounter, are fundamentally important to disease dynamics [75]. Some factors such as environmental and climatic determinants of transmission are readily available from RS sources [93], so RS techniques are particularly useful for the study of viral, bacterial and parasitic infections. These rely on intermediate hosts to complete their life cycles or on vectors for their spread, which are particularly vulnerable to changes in environmental factors such as temperatures, humidity and vegetation. Therefore, RS-derived knowledge of the limits of the distribution of, for example, the snail hosts of schistosomiasis or the mosquitoes that carry malaria, enables the disease risks to be estimated with a good level of accuracy [94]. Many countries have adopted RS techniques for disease control [12,22,52,67,95-104], and great strides have been made. But there are several issues that need to be considered for more effectively applying RS techniques in disease control, including the continuously easy and rapid availability of RS data in a timely manner; training of persons on RS data gathering, processing, modeling and interpretation of results; and close collaboration among researchers in different fields from the very beginning of research projects [80,105].

Beside RS, spatial data analysis and GIS are also important techniques and they are always used together [22,23,97]. But they do have their own features and lay different emphasis on studying disease control, so they can be studied separately. In this study, we have resisted the temptation to discuss them together but instead have focused on RS studies alone to maintain clarity. We have provided the first complete review on applying RS techniques in disease control in China and have identified the possible problems and challenges, hoping to shed light on future research directions on applying RS techniques to improve human health.

## Competing interests

The authors declare that they have no competing interests.

## Authors’ contributions

ZZJ and JQW defined the research theme. GJ, WZL and YBD designed literature search methods. All authors have contributed to read the selected papers, draft and revised the manuscript.
